# Amazon Amandaba—Sociodemographic Factors, Health Literacy, Biochemical Parameters and Self-Care as Predictors in Patients with Type 2 Diabetes Mellitus: A Cross-Sectional Study

**DOI:** 10.3390/ijerph20043082

**Published:** 2023-02-09

**Authors:** Victória Brioso Tavares, Aline Lobato de Farias, Amanda Suzane Alves da Silva, Josiel de Souza e Souza, Hilton Pereira da Silva, Maria do Socorro Castelo Branco de Oliveira Bastos, João Simão de Melo-Neto

**Affiliations:** 1 Postgraduate Program in Health, Environment and Society in the Amazon, Institute of Health Sciences, Federal University of Pará (UFPA), Belém 66050-160, Brazil; 2 Faculty of Physiotherapy and Occupational Therapy (FFTO), Federal University of Pará (UFPA), Belém 66075-110, Brazil

**Keywords:** diabetes mellitus, glycemic control, health literacy, self-care, primary health care

## Abstract

Background: Health literacy (HL) and its domains (functional, critical, and communicative) appear to be related to self-care adherence in people with type 2 diabetes mellitus (DM2). This study aimed to verify if sociodemographic variables are predictors of HL, if HL and the sociodemographic factors affect biochemical parameters together, and if HL domains are predictors of self-care in DM2. Methods: We used the baseline assessment data from 199 participants ≥ 30 years in the project, “Amandaba na Amazônia: Culture Circles as a Strategy to Encourage Self-care for DM in Primary Health Care,” which took place in November and December 2021. Results: In the HL predictor analysis, women (*p* = 0.024) and higher education (*p* = 0.005) were predictors of better functional HL. The predictors of biochemical parameters were: glycated hemoglobin control with low critical HL (*p* = 0.008); total cholesterol control with female sex (*p* = 0.004), and low critical HL (*p* = 0.024); low-density lipoprotein control with female sex (*p* = 0.027), and low critical HL (*p* = 0.007); high-density lipoprotein control with female sex (*p* = 0.001); triglyceride control with low Functional HL (*p* = 0.039); high levels of microalbuminuria with female sex (*p* = 0.014). A low critical HL was a predictor of a lower specific diet (*p* = 0.002) and a low total HL of low medication care (*p* = 0.027) in analyses of HL domains as predictors of self-care. Conclusion: Sociodemographic factors can be used to predict HL, and HL can predict biochemical parameters and self-care.

## 1. Introduction

Health Literacy (HL) was coined in 1970 [1] and was initially defined as the ability to deal with words and numbers in a medical setting. However, the concept of HL has evolved, and it is now considered to encompass a variety of social, personal, and cognitive skills required to obtain, process (critical thinking), and understand the information in the health context [2,3,4,5,6,7].

According to Nutbeam [8] HL has three dimensions: functional HL (FHL)—the ability to read and write; communicative HL (CHL)—the ability to absorb and apply the information obtained; and critical HL (CrHL)—the analysis and deeper understanding of information for decision-making. These different dimensions can affect the patient’s autonomy and ability to use health information, which affects self-care and treatment adherence decision-making [9]. Low levels of HL can lead to harmful health practices [10], including less knowledge, management, and self-care, resulting in lower medication adherence and increased hospitalization and mortality, especially in those with non-communicable diseases (NCD) [11,12,13].

The Test of Functional Health Literacy in Adults [14], the Rapid Estimate of Adult Literacy in Medicine [15], and the Newest Vital Sign [16] are among the HL assessment instruments that seek to measure HL more objectively and only assess specific dimensions. However, more recent instruments, including the European Health Literacy Survey Questionnaire [17], the Health Literacy Questionnaire [18], and the 14-item health literacy scale (HLS-14) [19] consider the multidimensionality of the HL and measure self-reported HL.

Diabetes mellitus (DM) is an NCD that requires understanding a wide range of clinical recommendations and information to manage the disease, including self-care activities, such as healthy eating, physical activity, adherence to prescribed medications, and foot care. Diabetes affects approximately 463 million people globally (aged between 20 and 70 years). Brazil ranks sixth among the top ten countries or territories in terms of the number of adults (20–79 years) with diabetes (15.7 million in 2021) [20,21].

The most common methods for assessing self-care behaviors are questionnaires on the adoption of behaviors during a specific period, typically 24 h [22], 1 week [23], or 1 month [24], or the frequency of specific self-care behaviors during the previous week [25].

In Brazil, no studies have reported comprehensive data on HL among people with type 2 diabetes mellitus (DM2). Most studies use questionnaires that assess only one component of HL, making it difficult to understand the magnitude of HL. Nevertheless, previous studies have reported that an insufficient level of some HL domains seems to be associated with low adherence to self-care behavior in people with DM2, especially regarding glycemic monitoring and control [26,27,28]. Although few studies have used specific instruments to evaluate the direct influence of HL on different aspects of self-care in DM2, approximately 65% of people with DM2 have been reported to have low HL [29]. People living in the northern region of Brazil and older adults with DM2, whom the Unified Health System assists, seem to have low HL [30].

Because the World Health Organization defines HL as a social determinant of health mediated by cultural and situational demands [31], it is necessary to understand how HL relates to sociodemographic factors, and due to the context of the DM, how it relates to biochemical parameter control and self-care.

Thus, this study aimed to determine if sociodemographic factors and HL are predictors of biochemical parameters (glycated hemoglobin, triglycerides, total cholesterol, high-density lipoprotein (HDL), low-density lipoprotein (LDL), non-high-density lipoprotein cholesterol (NHDL), and microalbuminuria) and whether HL and its domains are predictors of specific self-care behaviors in patients with DM2 in Brazil. We hypothesized that other sociodemographic variables are essential for HL diagnosis and that HL influences biochemical parameter control and self-care behavior maintenance.

## 2. Materials and Methods

### 2.1. Study Design

This was a cross-sectional observational study with descriptive and inferential statistics.

### 2.2. Setting and Period of Study

The study used data from the participants’ baseline assessments in the project “Amandaba na Amazônia: Culture Circles as a Strategy to Encourage Self-care for DM in Primary Health Care”, which took place in November and December 2021. The project was conducted in the Belém-Pará setting of the Unified Brazilian Health System, using a survey of patients with DM2 registered in the Family Health Strategies (FHS) in two administrative health districts of the municipality, district 1: Guamá, and district 2: Bengui. 

### 2.3. Population

The study population consisted of individuals with DM2 aged 30 years or older.

### 2.4. Eligibility Criteria

The inclusion criteria were patients aged ≥30 years with DM2 that were registered and had been followed up for at least 1 year in one of the eight selected FHS units. In addition, the participants had to be able to read and have adequate or corrected self-reported hearing and visual acuity to understand the study. 

### 2.5. Sampling

Probabilistic random sampling, through a drawing based on the survey of the defined population, was used to select patients. 

### 2.6. Sample

**Figure 1:**
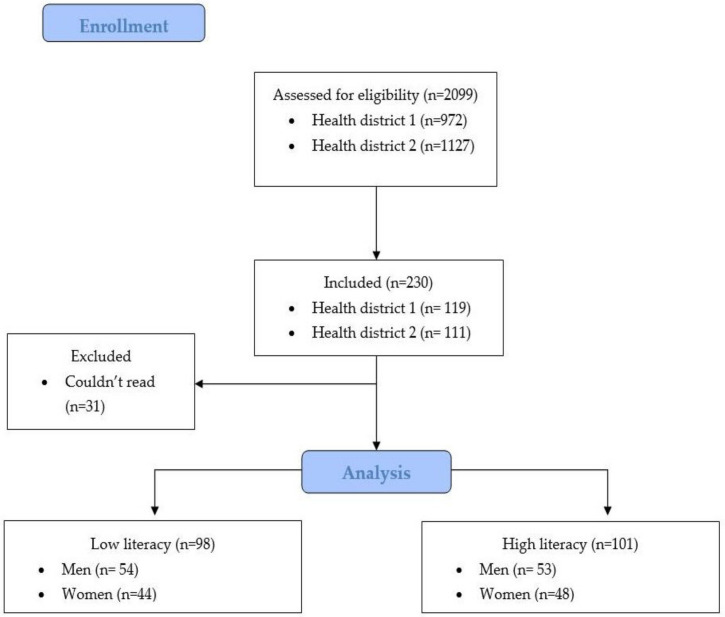
Flow diagram of the selection and distribution of individuals in the groups.

The sample size was calculated using the Gpower 3.1 software (HUU, Dusseldorf, Germany), based on the variable “Hb1Ac (glycated hemoglobin)” in the study by Rodrigues et al. [32] who compare HL in adults and older adults with diabetes between health units in two municipalities in São Paulo, Brazil, using the 14-item health literacy scale [18,33,34]. This presented an odds ratio of 4.455 and a *p* value < 0.05, indicating a minimum sample of 79 participants (low literacy = 34; high literacy = 45). This was based on the N2/N1 allocation ratio of 1.33, a proportion p2 of 0.125, β error probability of 0.8, α error probability of 0.05. Our initial sample consisted of 230 individuals, of whom 199 were selected based on the eligibility criteria (Figure 1).

### 2.7. Data Collection and Variables

An individual standardized questionnaire was used to collect data, which included the following sociodemographic variables: sex, age, race, education (number of years of formal education), HL, and per capita income; and health-related and clinical characteristics: duration of DM2 diagnosis (years), perception of general health, smoking and alcohol consumption, number of consultations in the previous year, private health insurance, systemic arterial hypertension (SAH), physical activity, and peripheral neuropathy. Physical activity was assessed using the Brazilian national self-assessment of health status survey, which categorizes it as sedentary for those who identify with the answer options “sitting most of the day” and “does not walk much during the day”, regular: “carries light weights or climbs stairs frequently or exercises regularly” and “carries heavy weights or exercises regularly”, and irregular: “walks or stands a lot during the day, but does not carry or lift weights regularly” was replaced by “irregular physical activity” [35]. The Michigan Neuropathy Screening Instrument (MSNI) was used to assess peripheral neuropathy; MSNI values ≤5 = no neuropathy, ≥5.5 = neuropathy [36]; biochemical parameters: triglycerides (normal: 36–149; high ≥150 mg/dL), total cholesterol (normal: 87–189; high ≥190 mg/dL), HDL (normal ≥40; low: 20–39 mg/dL), LDL (normal: 15–99; high ≥100 mg/dL), NHDL (normal: 59–129; high ≥130 mg/dL), HbA1c (normal: 4.8–6.9; high ≥7%), and microalbuminuria (low <30; normal: 30–300; high >300 mg/g), were obtained through laboratory tests.

HL was assessed using the 14-item health literacy scale developed by Suka et al. [18] and validated in Brazilian Portuguese by Batista et al. [34]. It is one of the few instruments with a fair quality assessment that considers the expanded concept of a relatively short assessment, and has been translated and validated into Brazilian Portuguese. It is scored on a 5-point Likert scale, with responses ranging from “totally agree” to “strongly disagree”, organized into three dimensions: functional (five items), communicative (five items), and critical (four items), according to the theoretical model of HL proposed by Nutbeam [8]. The total score ranges from 14 to 70, and higher average scores for each item are associated with higher literacy, except in the functional dimension, where the result is inverted. Based on the average of the total score of the participants, the classification of HL was divided into low (<46) and high (≥46), representing a high or low ability to access, absorb and apply, and understand health information in accordance with the HLS-14 specific domains and overall score [8,34]. The Brazilian version of the Diabetes Self-Care Activities Questionnaire (QAD) [37] was used to assess self-care, which covered five aspects of the diabetes treatment regimen: general diet, specific diet, physical activity, glycemic monitoring, foot care, and medication. These dimensions represent various diabetes treatment activities performed independently by patients, with questions about the frequency of activities performed in the previous 7 days and their agreement with the doctor’s or other health professional’s prescription. Thus, a score of 7 means ideal self-care adherence, while a score of <3 indicates minor care. Each aspect’s median number of days is examined.

The invitation signature of the consent form and the administration of the questionnaires and laboratory tests were all done at the participant’s home during a previously scheduled visit. All questionnaires were administered personally by 12 researchers who had been trained on a protocol for approaching and using the instruments.

### 2.8. Primary Outcomes

The primary outcomes were defined according to the following objectives: (1) HL, (2) biochemical parameters, and (3) self-care.

### 2.9. Bias

The study design made it susceptible to non-response bias, which was attempted to be minimized through prior scheduling of visits and the benefit of returning the results of laboratory tests to participants to encourage participation in the study, so no questionnaire was incomplete. However, in our study direct contact was made with the participant drawn from a list, with each participant being replaced by the next in sequence, so only those who accepted the invitation after the drawing were included. The selection bias was minimized by obtaining a sample from a defined population and reporting the population selection steps and recruitment/inclusion criteria to minimize the unwarranted generalization of the findings.

### 2.10. Statistical Analysis

Descriptive statistical analysis was performed to calculate the frequency (absolute and relative), mean and standard deviation (parametric), or medians with interquartile range (IQR, non-parametric) as measures of central and dispersion tendency, respectively. The data underwent the Kolmogorov–Smirnov normality test, Pearson’s chi-square test, Fisher’s exact test, and the Mann–Whitney U test. A Z_crit_ value of ≥1.96 was considered for the post hoc residual adjustment test Multiple linear regression (for microalbuminuria and QAD) and multivariate logistic regression models with an enter approach were developed to delineate the relationships between the continuous and categorical variables, and the various domains of HL and self-care and between HL and laboratory tests. The entry criteria for the final logistic regression model were a *p*-value of <0.20 [38], the absence of multicollinearity (Tolerance >0.10, VIF <10) and the normality of residuals (Durbin–Watson: 1.5–2.5) were analyzed for both linear and logistic regressions. A *p*-value of < 0.05 was considered statistically significant. The IBM SPSS Statistics 26.0 software was used for the analysis.

## 3. Results

### 3.1. Analysis of The Sociodemographic, Health-Related, and Clinical Characteristics between Groups

Table 1 shows the general characteristics of the participants and their attributes according to the HLS-14. In terms of sociodemographic variables, men were predominant (53.8%), and those above 60 years old (54.3%), black and brown (88.4%), high FHL (56.3%), CHL (50.3%), and CrHL (56.8%), with a mean education of 7.6 years and a mean per capita income of BRL 482.4, with the majority receiving between BRL 300 and 600 (38.7%). Regarding the health-related and clinical characteristics, there was a predominance of a common perception of general health status (50.3%), a diagnosis time of 5–10 years (38.7%), one to five consultations per year (54.3%), no private health insurance (88.4%), non-smokers (50.3%), alcohol consumers (67.3%), those with irregular physical activity (46.2%), SAH (68.3%), and peripheral neuropathy (66.3%). Among the biochemical parameters, there was a predominance of high levels of total cholesterol (50.8%), LDL (61.8%), NHDL (63.3%), triglycerides (63.8%), and glycated hemoglobin (83.4%), with levels within the parameters indicated for HDL (52.8%) and microalbuminuria (71.4%). In the QAD, the averages were low (less than 3.5 days) for the following domains: general diet (mean: 3.0, SD: 2.5), specific diet (mean: 1.5, SD: 0.9), physical activity (mean: 1.6, SD: 2.2), and glycemic monitoring (mean: 1.0, SD: 1.9), and high for the domain’s foot care (mean: 3.7, SD: 2.2), and medication (mean: 4.4, SD: 1.8). 

Differences were observed between the high and low HL groups for the following variables: FHL, CHL, and CrHL (*p* < 0.0001), with a predominance of high HL in all domains in G2, diagnosis time (*p* = 0.005), with a predominance of high HL diagnosed between 11 and 20 years, private health insurance (*p* = 0.025), and high HL among those with private health insurance. In the QAD, G2 scored higher in the foot care domain (*p* = 0.009) and medication domain (*p* = 0.013).

### 3.2. Sociodemographic Characteristics as Predictors of Health Literacy

In the univariate analysis (Table 2), education (*p* = 0.058) and per capita income (*p* = 0.092) had the lowest *p*-values for inclusion in the predictor model of total literacy, and for functional literacy, sex (*p* = 0.039) and education (*p* = 0.005). No variable provided the necessary value for analyzing the effect on communicative literacy; only per capita income provided the cut-off value (*p* = 0.069) in critical literacy. 

There was no multicollinearity between the selected variables. Multivariate analysis showed that being a woman (*p* = 0.024) and having a higher level of education (*p* = 0.005) were predictors of better functional literacy.

### 3.3. Sociodemographic Characteristics and Health Literacy as Predictors of Biochemical Parameters

The final analysis included 199 participants (Table 3). In the univariate analysis, the variables that presented the minimum *p*-value for inclusion in the predictor model of glycated hemoglobin were age (*p* = 0.017), critical literacy (*p* = 0.011), and per capita income (*p* = 0.066); for total cholesterol, sex (*p* = 0.003), age (*p* = 0.092), and critical literacy (*p* = 0.024); for LDL, sex (*p* = 0.022), age (*p* = 0.112), and critical literacy (*p* = 0.007); for HDL, sex (*p* = 0.000), age (*p* = 0.000), education (*p* = 0.197), and functional literacy (*p* = 0.082); for NHDL, sex (*p* = 0.066), age (*p* = 0.120), and critical literacy (*p* = 0.106); for triglycerides, functional literacy only (*p* = 0.005), and for microalbuminuria sex (*p* = 0.032 and *p* = 0.011), age (*p* = 0.028), education (*p* = 0.179), functional literacy (*p* = 0.130 and *p* = 0.192), communicative literacy (*p* = 0.026), and per capita income (*p* = 0.163 and *p* = 0.089).

There was no multicollinearity between the selected variables. Multivariate analysis showed that low critical literacy was a predictor of glycated hemoglobin control (*p* = 0.008); female sex (*p* = 0.004) and low critical literacy (*p* = 0.024) were predictors of total cholesterol control; the same was found for LDL, female sex (*p* = 0.027), and low critical literacy (*p* = 0.007). Only women predicted HDL control (*p* = 0.001), and low functional literacy predicted triglyceride control (*p* = 0.039). Women were predictors of high microalbuminuria levels (*p* = 0.014).

### 3.4. Health Literacy as Predictors of Self-Care

The final analysis included 199 participants (Table 4). All assumptions of the multiple linear regression were observed; among the variables, low critical literacy was a predictor of a lower specific diet (*p* = 0.002) and low total literacy of minor care with medication (*p* = 0.027). There were no effects of literacy on general diet, physical activity, foot care, and blood glucose monitoring.

## 4. Discussion

In this study we aimed to investigate the relationship between HL, sociodemographic factors, and because of the context of DM, the control of biochemical parameters and self-care in 199 patients with DM2 who were randomly selected from two health districts of a municipality in the northern region of Brazil.

According to the HLS-14, 50.7% of the people in our sample had a high total level of HL. The assessment of HL in patients with DM2 varied greatly across countries [39]. Previous studies in Brazil using the HLS-14 reported percentages of adequate HL ranging from 51.4 to 56.2% [40,41,42], and low HL ranging from 33.8% to 51.6%, specifically among public health service users [32,38]. A higher level of education and being a woman were identified as independent factors of a high FHL level based on sociodemographic information. This finding is consistent with Nutbeam’s functional HL definition of “basic skills in reading and writing to enable individuals to function effectively in everyday situations” [8].

Findings on the association between sex and HL are inconsistent worldwide. The disparities in HL between men and women may be related to the fact that women outperformed men in basic educational indicators, including the “adjusted rate of net school attendance to the initial and final grades of elementary school”, and the “adjusted rate of net school attendance in the high school for 15- to 17-year-olds” between 2016 and 2019 in Brazil, particularly in the Northern region [43]. Furthermore, it may be related to women having a greater familiarity with navigating the health system to deal with health issues, which may provide more opportunities to build their knowledge base [44].

In the analysis of the biochemical control predictors, women were independent predictors of the control of total cholesterol, LDL, and HDL levels, but a predictor of levels above the control of microalbuminuria. Women tend to eat healthier than men, consuming more fruit and vegetables and less meat. However, women may be more sedentary and have a lower success rate of glucose-lowering therapy [45], thereby increasing the risk of dual therapy failure. 

In contrast to the findings of previous studies that reported that women with DM2 had significantly higher glycated hemoglobin levels [46,47] and poorer glycemic control than men, our findings indicate the opposite. Although data from high-income countries indicate that women are less likely than men to receive the care recommended by guidelines, to adhere to glycemia-lowering therapy, and to meet treatment targets for glycemia and lipids and that women are frequently reported as hurting diabetes self-management [48], studies of the indicators of the line of care for people with diabetes in Brazil reported that in 2019 women used the Popular Pharmacy Program more often to obtain medication (53.4%), had a higher proportion of medical assistance in the past year (81.0%), had their last appointment for DM follow-up at a PHC center (51.1%), and were hospitalized less often due to DM or complications (13.1%) [21]. Additionally, the regulation of glucose homeostasis, treatment response, and psychological factors [46] may contribute to the difference between men’s and women’s biochemical control.

Lack of control in biochemical parameters was frequent in both groups of our sample, which may have affected the result that low CrHL was an independent predictor of glycated hemoglobin and total and LDL cholesterol control, and that low FHL was an independent predictor of triglyceride control. Considering the HL concept, it is arguable that HL mediates the control of biochemical parameters indirectly through health decision-making, as the CrHL requires “skills to critically analyze and use the information to exert control over life events and situations” [49]. However, the findings are still inconsistent because HL is significantly more associated with behaviors such as diet, physical activity, and medication use. Therefore, it may not be a direct determinant of biochemical parameters as the control involves a collection of physiological factors that are unique to each individual and can interfere with the effect of adopting general health practices. In addition, patients with higher HL may have similar odds of achieving a clinical goal as those with low HL, regardless of their baseline HL levels. They may engage in self-care activities, including self-monitoring of blood glucose [50].

Despite this, low HL was a predictor of minimal medication care, and low CrHL was a predictor of less adherence to a specific diet. A meta-analysis [51] revealed a weak relationship between HL and medication adherence, possibly due to other factors including adherence determinants. However, in our study this was the only concept of the QAD for which the total score of the questionnaire was decisive, assuming that the set of HL domains influences self-care. The QAD-specific diet concept reflects self-care activities that require a more complex elaboration of nutritional knowledge [37], corroborating the CrHL concept, which necessitates empowering patients to assimilate and implement health-related information and knowledge [52,53,54].

Brazil has the most publications on HL (20%) among South American countries, with most studies focusing on the functional aspect of HL. This study contributes to the literature by assessing the other dimensions of the HL [35].

It is noteworthy that because of the nature of the study, the results represent a specific population at a specific time. Therefore, the generalization of these findings to other sociodemographic and cultural contexts may be limited. Investigation of the potential interactions of other specific clinical aspects was beyond the objective of this study, but their relationship with biochemical parameter control can be considered a limitation. Finally, our study highlights the need for national and regional studies on HL and DM, particularly in terms of self-care activities and their impact on the control of clinical parameters.

## 5. Conclusions

In this study, patients who took longer to get diagnosed had private health insurance took better care of their feet, received regular medications, and had higher total HL. We found that women and a higher level of education were the sociodemographic variables that predicted better functional HL. A low critical HL level was a predictor of high value of glycated hemoglobin, total cholesterol, and LDL levels. Women were predictors of high value of total cholesterol, HDL, LDL, and a high level of microalbuminuria control. Only low functional HL was found to be a predictor of triglyceride level. In this DM2 population, only critically low HL was a predictor of a lower specific diet, and low total HL was a predictor of less medication care. 

## Figures and Tables

**Table 1:** General characteristics of participants and characteristics according to low (G1) and high (G2) literacy levels for HLS-14.

	Total	G1 n = 98 (49.3%)	G2 n = 101 (50.7%)	χ2 (or Fisher’s Exact Test)/Mann–Whitney	*p*-Value
Sociodemographic variables					
Sex					
Female	92 (46.6%)	44 (44.9%)	48 (47.5%)		
Male	107 (53.8%)	54 (55.1%)	53 (52.5%)	0.138 ^a^	0.777
Age (years)	62 (IQR: 15)	62 (IQR: 15)	61 (IQR: 15)	4924 ^c^	0.951
30–40	6 (3.0%)	3 (3.1%)	3 (3.1%)		
41–50	24 (12.1%)	12 (12.2%)	12 (11.9%)		
51–60	61 (30.7%)	26 (26.5%)	35 (34.7%)		
≥61	108 (54.3%)	57 (58.2%)	51 (50.5%)	1.711 ^b^	0.643
Race/color					
Blacks and Browns	176 (88.4%)	88 (89.8%)	88 (87.1%)		
Whites	23 (11.6%)	10 (10.2%)	13 (12.9%)	0.346 ^a^	0.659
Education (years of study)	8 (IQR: 7.0, 8.2)	7 (IQR: 6.2, 7.8)	9 (IQR: 7.3, 8.9)	4191.5 ^c^	0.060
Functional HL					
Low	87 (43.7%)	64 (73.6%)	23 (26.4%)		
High	112 (56.3%)	34 (30.4%)	78 (69.6%)	36.571 ^a^	0.000 *
Communicative HL					
Low	99 (49.7%)	68 (68.7%)	31 (31.3%)		
High	100 (50.3%)	30 (30.0%)	70 (70.0%)	29.790 ^a^	0.000 *
Critical HL					
Low	86 (43.2%)	62 (72.1%)	24 (27.9%)		
High	113 (56.8%)	36 (31.9%)	77 (68.1%)	31.629 ^a^	0.000 *
Per capita income (Reais)	400 (IQR: 435.0, 529.8)	367 (IQR: 383.4, 498.2)	413 (IQR: 447.6, 597.9)	4891.5 ^c^	0.171
<300	72 (36.2%)	36 (36.7%)	36 (35.6%)		
300–600	77 (38.7%)	43 (43.9%)	34 (33.7%)		
601–1000	35 (17.6%)	13 (13.3%)	22 (21.8%)		
>1000	15 (7.5%)	6 (6.1%)	9 (8.9%)	3.922 ^a^	0.274
Health-related and clinical characteristics					
Perception of general health status					
Very good	16 (8.0%)	6 (6.1%)	10 (9.9%)		
Good	59 (29.6%)	28 (28.6%)	31 (30.7%)		
Regular	100 (50.3%)	49 (50%)	51 (50.5%)		
Bad	15 (7.5%)	7 (7.1%)	8 (7.9%)		
Very bad	9 (4.5%)	8 (8.2%)	1 (1.0%)	6.666 ^b^	0.152
Diagnosis time (years)	8 (IQR: 11)	6 (IQR: 10.25)	10 (IQR: 10)	4303 ^c^	0.111
<5	53 (26.6%)	30 (30.6%)	23 (22.8%)		
5–10	77 (38.7%)	39 (39.8%)	38 (37.6%)		
11–20	46 (23.1%)	14 (14.3%) #	32 (31.7%) #		
21–30	17 (8.5%)	9 (9.2%)	8 (7.9%)		
>30	6 (3.0%)	6 (6.1%)	0 (0%)	14.134 ^b^	0.005 *
Consultations in past year					
0	21 (10.6%)	11 (11.2%)	10 (9.95%)		
1–5	108 (54.3%)	50 (51.0%)	58 (57.4%)		
>5	70 (35.2%)	37 (37.8%)	33 (32.7%)	0.824 ^a^	0.655
Private health insurance					
Yes	23 (11.6%)	6 (6.1%) #	17 (16.8%) #		
No	176 (88.4%)	91 (93.9%)	84 (83.2%)	5.158 ^a^	0.025 *
Smoking consumption					
Non-smokers	100 (50.3%)	42 (42.9%)	58 (57.4%)		
Ex-smoker	87 (43.7%)	48 (49%)	39 (38.6%)		
Smoker	12 (6.0%)	8 (8.2%)	4 (4%)	4.78 ^a^	0.093
Alcohol consumption					
Yes	134 (67.3%)	68 (69.4%)	66 (65.3%)		
No	65 (32.7%)	30 (30.6%)	35 (34.7%)	0.369 ^a^	0.550
Physical activity					
Sedentary	69 (34.7%)	32 (32.7%)	37 (36.6%)		
Irregular	92 (46.2%)	48 (49%)	44 (43.6%)		
Regular	38 (19.1%)	18 (18.4%)	20 (19.8%)	0.596 ^a^	0.748
SAH					
Yes	136 (68.3%)	70 (71.4%)	66 (65.3%)		
No	63 (31.7%)	28 (28.6%)	35 (34.7%)	0.850 ^a^	0.366
Peripheral neuropathy					
Present	132 (66.3%)	70 (71.4%)	62 (61.4%)		
Absent	67 (33.7%)	28 (28.6%)	39 (38.6%)	2.246 ^a^	0.177
Biochemical parameters					
Total cholesterol (mg/dL)					
87–189	98 (49.2%)	50 (51%)	48 (47.5%)		
≥190	101 (50.8%)	48 (49%)	51.3 (52.5%)	0.243 ^a^	0.671
HDL (mg/dL)					
20–39	94 (47.2%)	44 (44.9%)	50 (49.5%)		
≥40	105 (52.8%)	54 (55.1%)	51 (50.5%)	0.424 ^a^	0.571
LDL (mg/dL)					
15–99	76 (38.2%)	36 (36.7%)	40 (39.6%)		
≥100	123 (61.8%)	62 (63.3%)	60.1 (60.4%)	0.173 ^a^	0.771
Non-HDL (mg/dL)					
59–129	73 (36.7%)	40 (40.8%)	33 (32.7%)		
≥130	126 (63.3%)	58 (59.2%)	68 (67.3%)	1.420 ^a^	0.243
Triglycerides (mg/dL)					
36–149	72 (36.2%)	42 (42.9%)	30 (29.7%)		
≥150	127 (63.8%)	56 (57.1%)	71 (70.3%)	3.727 ^a^	0.057
Glycated hemoglobin (mg/dL)					
4.8–6.9%	33 (16.6%)	17 (17.3%)	16 (15.8%)		
≥7%	166 (83.4%)	81 (82.7%)	85 (84.2%)	0.081 ^a^	0.850
Microalbumi-nuria (Creatinina mg/g)					
<30	142 (71.4%)	71 (72.4%)	71 (70.3%)		
30–300	41 (20.6%)	18 (18.4%)	23 (22.8%)		
>300	16 (8.0%)	9 (9.2%)	7 (6.9%)	0.815 ^a^	0.705
QAD					
Geral diet (days)	3 (IQR: 5)	3 (IQR: 3.25)	3 (IQR: 5)	4896 ^c^	0.894
Specific diet (days)	1 (IQR: 1)	1 (IQR: 1)	1 (IQR: 1)	4875 ^c^	0.848
Physical activity (days)	0 (IQR: 4)	0 (IQR: 4)	0 (IQR: 4)	4893 ^c^	0.879
Foot care (days)	4 (IQR: 3)	3 (IQR: 3)	5 (IQR: 5)	3908.5 ^c^	0.009 *
Medication	5 (IQR: 1)	5 (IQR: 2)	5 (IQR: 0)	4037.5 ^c^	0.013 *
Glycemic monitoring (days)	0 (IQR: 1)	0 (IQR: 1)	0 (IQR: 1)	4891.5 ^c^	0.867

^a^ χ^2^ Pearson (two-tailed); ^b^ Fisher’s exact test (two-tailed); ^c^ Mann–Whitney U test (two-tailed). * Significant values by the post hoc residual adjustment test with Z_crit_ ≥ 1.96. # Adjusted residual post hoc tests with Z_crit_ ≥ 1.96.

**Table 2:** Univariate and multivariate analysis of sociodemographic characteristics as predictors of health literacy according to the HLS-14.

	N	OR ^1^	CI 95% ^1^	*p*-Value ^1^	OR ^2^	CI 95% ^2^	*p*-Value ^2^
TOTAL HL							
Sex	199	0.90	0.51, 1.57	0.710			
Age	199	1.00	0.97, 1.02	0.884			
Race/color	199	1.30	0.54, 3.12	0.557			
Education	199	1.07	0.98, 1.14	0.058 ^#^	0.93	0.87, 1.00	0.070
Per capita income	199	1.00	1.00, 1.00	0.092 ^#^	0.99	0.99, 1.00	0.110
FUNCTIONAL HL							
Sex	199	1.82	1.03, 3.22	0.039 ^#^	1.96	1.09, 3.52	0.024 *
Age	199	1.00	0.97, 1.03	0.728			
Race/color	199	0.80	0.33, 1.96	0.638			
Education	199	1.10	1.02, 1.18	0.008 ^#^	1.11	1.03, 1.19	0.005 *
Per capita income	199	1.00	0.99, 1.00	0.555			
COMMUNICATIVE HL							
Sex	199	0.83	0.47, 1.45	0.526			
Age	199	0.98	0.95, 1.01	0.214			
Race/color	199	0.73	0.30, 1.76	0.491			
Education	199	1.00	0.94, 1.07	0.856			
Per capita income	199	1.00	0.99, 1.00	0.664			
CRITICAL HL							
Sex	199	0.86	0.49, 1.51	0.614			
Age	199	0.98	0.95, 1.01	0.225			
Race/color	199	1.23	0.51, 2.94	0.636			
Education	199	0.99	0.92, 1.06	0.885			
Per capita income	199	1.00	1.00, 1.00	0.069 ^#^			

^1^ univariate analysis; ^2^ multivariate analysis. **^#^** *p*-value < 0.20; * *p*-value < 0.05.

**Table 3:** Univariate and multivariate analysis of HL and sociodemographic characteristics as predictors of glycated hemoglobin, total cholesterol, LDL, HDL, non-HDL, triglycerides, and microalbuminuria parameters.

	N	OR ^1^	95% CI ^1^	*p*-Value ^1^	OR ^2^	95% CI ^2^	*p*-Value ^2^
GLYCATED HEMOGLOBIN							
Sex	199	1.20	0.56, 2.55	0.631			
Age	199	0.95	0.91, 0.99	0.017 ^#^	0.96	0.92, 1.00	0.071
Race/color	199	0.73	0.20, 2.61	0.629			
Education	199	0.95	0.86, 1.04	0.283			
Total literacy	199	0.89	0.42, 1.89	0.775			
Functional literacy	199	0.91	0.43, 1.94	0.826			
Communicative literacy	199	0.79	0.37, 1.68	0.547			
Critical literacy	199	0.36	0.16, 0.79	0.011 ^#^	0.32	0.14, 0.74	0.008 *
Per capita income	199	0.99	0.99, 1.00	0.066 ^#^	0.99	0.99, 1.00	0.071
TOTAL CHOLESTEROL							
Sex	199	2.40	1.36, 4.26	0.003 ^#^	0.41	0.23, 0.75	0.004 *
Age	199	0.97	0.95, 1.00	0.092 ^#^	0.98	0.95, 1.01	0.323
Race/color	199	0.93	0.39, 2.23	0.885			
Education	199	0.98	0.91, 1.05	0.614			
Total HL	199	0.86	0.49, 1.51	0.622			
Functional HL	199	0.79	0.45, 1.38	0.416			
Communicative HL	199	1.19	0.68, 2.09	0.524			
Critical HL	199	1.88	1.06, 3.31	0.029 ^#^	0.50	0.28, 0.91	0.024 *
Per capita income	199	1.00	0.99, 1.00	0.732			
LDL							
Sex	199	0.50	0.28, 0.90	0.022 ^#^	0.50	0.27, 0.92	0.027 *
Age	199	0.97	0.95, 1.00	0.112 ^#^	0.98	0.95, 1.01	0.328
Race/color	199	1.04	0.42, 2.54	0.921			
Education	199	0.99	0.92, 1.06	0.843			
Total HL	199	1.12	0.63, 2.00	0.677			
Functional HL	199	1.44	0.80, 2.59	0.215			
Communicative HL	199	0.83	0.46, 1.47	0.523			
Critical HL	199	0.45	0.25, 0.80	0.007 ^#^	0.43	0.24, 0.79	0.007 *
Per capita income	199	1.00	0.99, 1.00	0.545			
HDL							
Sex	199	0.35	0.19, 0.63	0.000 ^#^	0.34	0.18, 0.62	0.001 *
Age	199	0.32	0.17, 0.58	0.000 ^#^	1.02	0.99, 1.05	0.071
Race/color	199	1.25	0.52, 2.98	0.615			
Education	199	1.04	0.97, 1.12	0.197 ^#^	1.06	0.98, 1.14	0.096
Total HL	199	1.20	0.68, 2.10	0.515			
Functional HL	199	1.65	0.93, 2.91	0.082 ^#^	1.64	0.89, 3.02	0.112
Communicative HL	199	0.83	0.47, 1.45	0.526			
Critical HL	199	1.05	0.60, 1.84	0.858			
Per capita income	199	1.00	0.99, 1.00	0.403			
Non-HDL							
Sex	199	0.58	0.32, 1.03	0.066 ^#^	0.60	0.33, 1.09	0.093
Age	199	0.97	0.95, 1.00	0.120 ^#^	0.98	0.95, 1.01	0.265
Race/color	199	1.09	0.44, 2.73	0.841			
Education	199	0.99	0.92, 1.06	0.844			
Total HL	199	0.70	0.39, 1.25	0.23			
Functional HL	199	1.08	0.60, 1.94	0.786			
Communicative HL	199	0.86	0.48, 1.53	0.621			
Critical HL	199	0.61	0.34, 1.10	0.106 ^#^	0.61	0.34, 1.11	0.112
Per capita income	199	1.00	1.00, 1.00	0.250			
TRIGLYCERIDES							
Sex	199	0.93	0.52, 1.67	0.833			
Age	199	0.98	0.95, 1.01	0.318			
Race/color	199	0.28	0.22, 1.56	0.288			
Education	199	1.04	0.97, 1.12	0.193 ^#^	1.03	0.95, 1.11	0.405
Total HL	199	0.56	0.31, 101	0.055 ^#^	0.71	0.36, 1.38	0.322
Functional HL	199	0.42	0.23, 0.77	0.005 ^#^	0.49	0.25, 0.96	0.039 *
Communicative HL	199	1.27	0.71, 2.28	0.406			
Critical HL	199	1.20	0.67, 2.17	0.529			
Per capita income	199	0.99	0.99, 1.00	0.142 ^#^	0.99	0.99, 1.00	0.077
MICROALBUMINURIA							
Sex							
30–300	199	2.16	1.07, 4.29	0.032 ^#^	1.77	0.82, 3.81	0.140
>300	199	4.60	1.41, 15.00	0.011^#^	4.74	1.37, 16.41	0.014 *
Age							
30–300	199	1.04	1.00, 1.08	0.028 ^#^	1.03	0.99, 1.08	0.070
>300		1.02	0.97, 1.08	0.281	1.00	0.94, 1.06	0.819
Race/color							
30–300	199	0.57	0.21, 1.51	0.263	0.59	0.20, 1.72	0.337
>300	199	1.77	0.21, 14.37	0.592	1.99	0.23, 17.39	0.530
Education							
30–300	199	0.96	0.88, 1.04	0.370	0.94	0.85, 1.03	0.237
>300	199	0.91	0.80, 1.04	0.179 ^#^	0.944	0.81, 1.09	0.440
Total HL							
30–300	199	0.78	0.38, 1.57	0.492			
>300	199	1.28	0.45, 3.64	0.636			
Functional HL							
30–300	199	0.56	0.27, 1.18	0.130 ^#^	0.48	0.21, 1.09	0.080
>300	199	2.03	0.70, 5.89	0.192 ^#^	2.30	0.72, 7.32	0.158
Communicative HL							
30–300	199	0.43	0.21, 0.90	0.026 ^#^	0.31	0.14, 0.69	0.311
>300	199	0.84	0.30, 2.37	0.844	0.90	0.30, 2.75	0.866
Critical HL							
30–300	199	0.78	0.38, 1.58	0.492			
>300	199	0.73	0.25, 2.12	0.565			
Per capita income							
30–300	199	1.00	1.00, 1.00	0.163 ^#^	1.00	0.99, 1.00	0.518
>300	199	1.00	1.00, 1.00	0.089 ^#^	1.00	0.99, 1.00	0.283

^1^ univariate analysis; ^2^ multivariate analysis. ^#^ *p*-value < 0.20; * *p*-value < 0.05.

**Table 4:** Multivariate linear regression analysis of literacy as predictors of self-care.

	β	t	*p*-Value
GENERAL DIET			
Constant	3.327	8.655	0.000
Total HL	0.548	1.108	0.269
Functional HL	-0.779	−1.775	0.078
Communicative HL	−0.206	−0.497	0.620
Critical HL	−0.094	−0.225	0.822
SPECIFIC DIET			
Constant	1.353	9.286	0.000
Total HL	−0.154	−0.823	0.412
Functional HL	−0.033	−0.200	0.841
Communicative HL	0.003	0.019	0.985
Critical HL	0.485	3.073	0.002 *
PHYSICAL ACTIVITY			
Constant	1.669	4.896	0.000
Total HL	0.009	0.022	0.983
Functional HL	−0.012	−0.032	0.975
Communicative HL	−0.041	−0.111	0.912
Critical HL	0.029	0.080	0.937
FOOT CARE			
Constant	3.193	9.637	0.000
Total HL	0.723	1.694	0.092
Functional HL	0.105	0.276	0.783
Communicative HL	0.093	0.261	0.795
Critical HL	0.076	0.211	0.833
MEDICATION			
Constant	4.276	15.278	0.000
Total HL	0.804	2.229	0.027 *
Functional HL	−0.093	−0.291	0.771
Communicative HL	−0.008	−0.025	0.980
Critical HL	−0.380	−1.255	0.211
BLOOD GLUCOSE MONITORING			
Constant	0.740	2.478	0.014
Total HL	0.079	0.205	0.838
Functional HL	0.315	0.924	0.356
Communicative HL	0.073	0.226	0.821
Critical HL	0.079	0.304	0.762

* *p*-value < 0.05.

## Data Availability

The data presented in this study are available on request from the corresponding author.
